# RNfuzzyApp: an R shiny RNA-seq data analysis app for visualisation, differential expression analysis, time-series clustering and enrichment analysis

**DOI:** 10.12688/f1000research.54533.2

**Published:** 2021-11-12

**Authors:** Margaux Haering, Bianca H Habermann

**Affiliations:** 1 Aix-Marseille University, CNRS, IBDM UMR 7288, The Turing Centre for Living systems, Marseille, 13009, France

**Keywords:** RNA-seq, data normalization, data visualization, differential expression analysis, time-series analysis, soft clustering, Mfuzz, R shiny

## Abstract

RNA sequencing (RNA-seq) is a widely adopted affordable method for large scale gene expression profiling. However, user-friendly and versatile tools for wet-lab biologists to analyse RNA-seq data beyond standard analyses such as differential expression, are rare. Especially, the analysis of time-series data is difficult for wet-lab biologists lacking advanced computational training. Furthermore, most meta-analysis tools are tailored for model organisms and not easily adaptable to other species.

With RNfuzzyApp, we provide a user-friendly, web-based R shiny app for differential expression analysis, as well as time-series analysis of RNA-seq data. RNfuzzyApp offers several methods for normalization and differential expression analysis of RNA-seq data, providing easy-to-use toolboxes, interactive plots and downloadable results. For time-series analysis, RNfuzzyApp presents the first web-based, fully automated pipeline for soft clustering with the Mfuzz R package, including methods to aid in cluster number selection, cluster overlap analysis, Mfuzz loop computations, as well as cluster enrichments.

RNfuzzyApp is an intuitive, easy to use and interactive R shiny app for RNA-seq differential expression and time-series analysis, offering a rich selection of interactive plots, providing a quick overview of raw data and generating rapid analysis results. Furthermore, its assignment of orthologs, enrichment analysis, as well as ID conversion functions are accessible to non-model organisms.

## Introduction

The development of next generation sequencing (NGS) methods has boosted the rapid generation of large datasets and RNA sequencing (RNA-seq) has become the standard for performing robust transcriptional profiling and thus quantifying gene expression in various contexts. Next to the comparison of two conditions, the generation of time-series RNA-seq data has become amenable and popular, allowing to monitor the gene expression dynamics over a process such as development, ageing or cancerogenesis. While web-based, user-friendly R shiny apps have become available recently for differential expression analysis and data visualization of RNA-seq data, ^1^^–^^7^ the analysis of time-series data within R remains largely command-line based and therefore challenging for bench scientists without programming knowledge.

We here present RNfuzzyApp, a user-friendly, web-based R shiny app with an intuitive user interface for the full workflows of differential expression analysis, as well as time-series analysis of RNA-seq data. RNfuzzyApp provides an interface for easy and fast data normalization and differential analysis using several methods, a variety of interactive plots for a quick overview of data and results, and an easy-to-use interface for the complete pipeline of time-series expression analysis using the fuzzy clustering algorithm Mfuzz. ^8^ In addition, RNfuzzyApp offers ID conversion, orthology assignment and enrichment analysis using gprofiler2. ^9^ We show the usability of RNfuzzyApp on two examples: an RNA-seq dataset of the ageing limb muscle of mouse, as well as developmental time-series RNA-seq data of the *Drosophila melanogaster* leg.

## Methods

### Implementation

RNfuzzyApp was built in R (V.4.0.4) using the Shiny framework. The app currently depends on the following R packages: *shiny, shinydashboard, shinycssloaders, shinythemes, shinyWidgets, shinyBS, rmarkdown, plotly, dplyr, RColorBrewer, utils, tidyr, devtools, cluster, DESeq2*, ^10^ *edgeR*, ^11^ *TCC*, ^12^ including *baySeq*, ^13^ *heatmaply*, ^14^ *gprofiler2*, ^9^ *Mfuzz*, ^8^ as well as the package *e1071.* As a basic feature and to allow users to upload any identifier for analysis, ID conversion is included, using the *gprofiler2* package.

### Operation

RNfuzzyApp can be launched locally from any computer with R (version 4.0.4 or higher) installed and will run in any web-browser. As RNfuzzyApp auto-installs all required R-packages, there exist no additional software requirements. Installation instructions are also available. All interfaces and plots of RNfuzzyApp are highly interactive, allowing users to visualize data in real-time as well as to interact efficiently with the data and plots.

### Workflows of RNfuzzyApp

**Figure 1.**
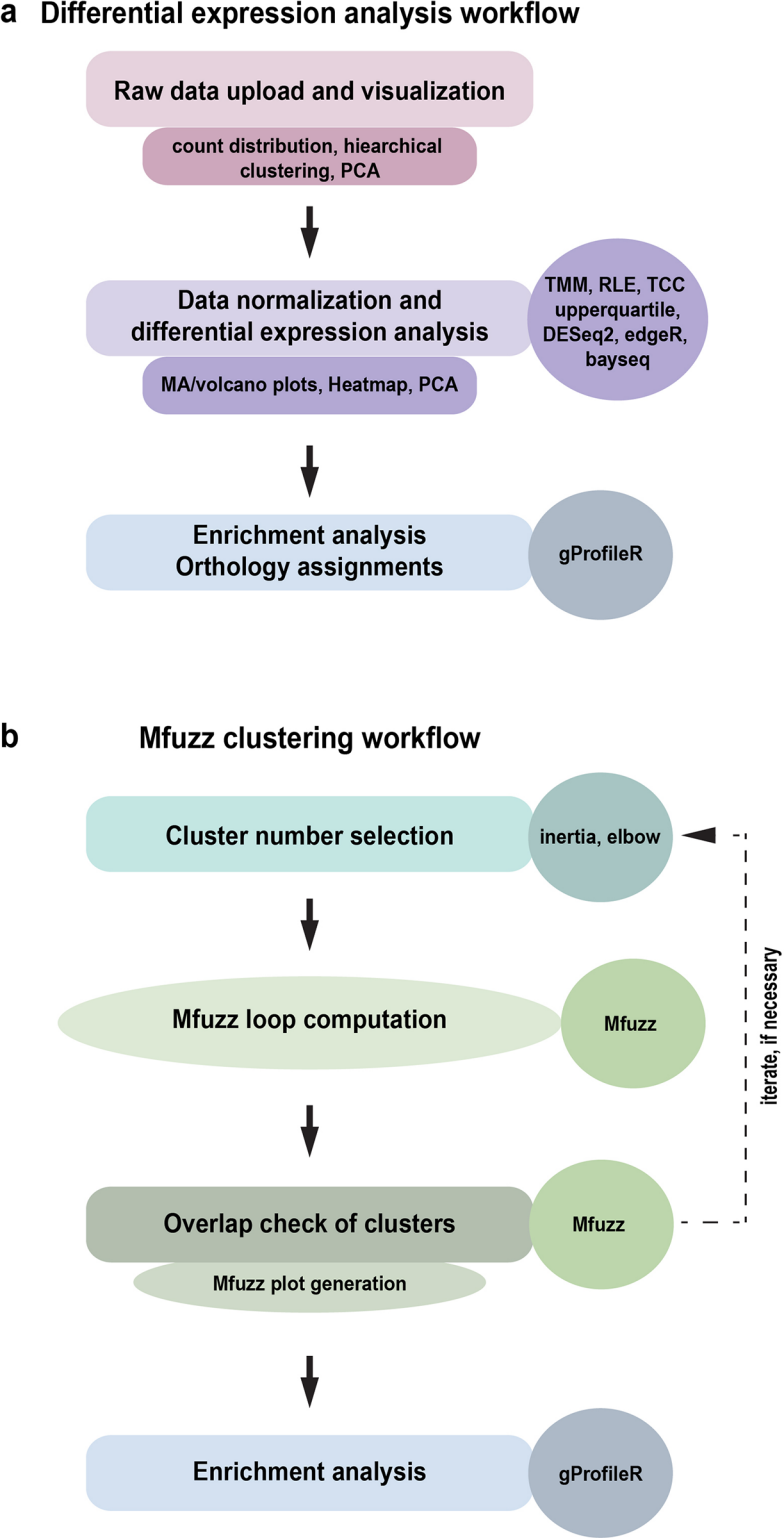
The two RNFuzzyApp analysis pipelines. (a) RNA-seq differential expression analysis workflow with the three main parts: data upload and visualization, data normalization and differential expression analysis, as well as enrichment analysis and the assignment of orthologous genes across species. The types of analyses are shown, as well as the various possible R programs provided for data analysis. (b) Mfuzz workflow for clustering of time-series RNA-seq data. The workflow includes the selection of cluster numbers, checking the overlap of Mfuzz clusters, loop calculations of Mfuzz, Mfuzz plot generation, as well as enrichment analysis of Mfuzz clusters.

The general workflow of RNfuzzyApp is shown in Figure 1. It can be divided into two independent parts: 1) a complete workflow for differential expression analysis of RNA-seq data ( Figure 1a); and 2) a complete workflow for the clustering of RNA-seq using the soft clustering algorithm Mfuzz ( Figure 1b).

### Differential expression analysis workflow of RNfuzzyApp

The differential expression analysis workflow of RNfuzzyApp can be divided in three main parts: the upload and visualization of the raw data; the normalization of the data and the differential expression analysis; and finally, enrichment analysis of results and orthology assignment ( Figure 1a). In each part, several options exist for visualizing the data and thus getting a first-hand impression of the quality of the data, as well as the filters that are applied.

*Data upload and visualization of raw gene expression data*

**Figure 2.**
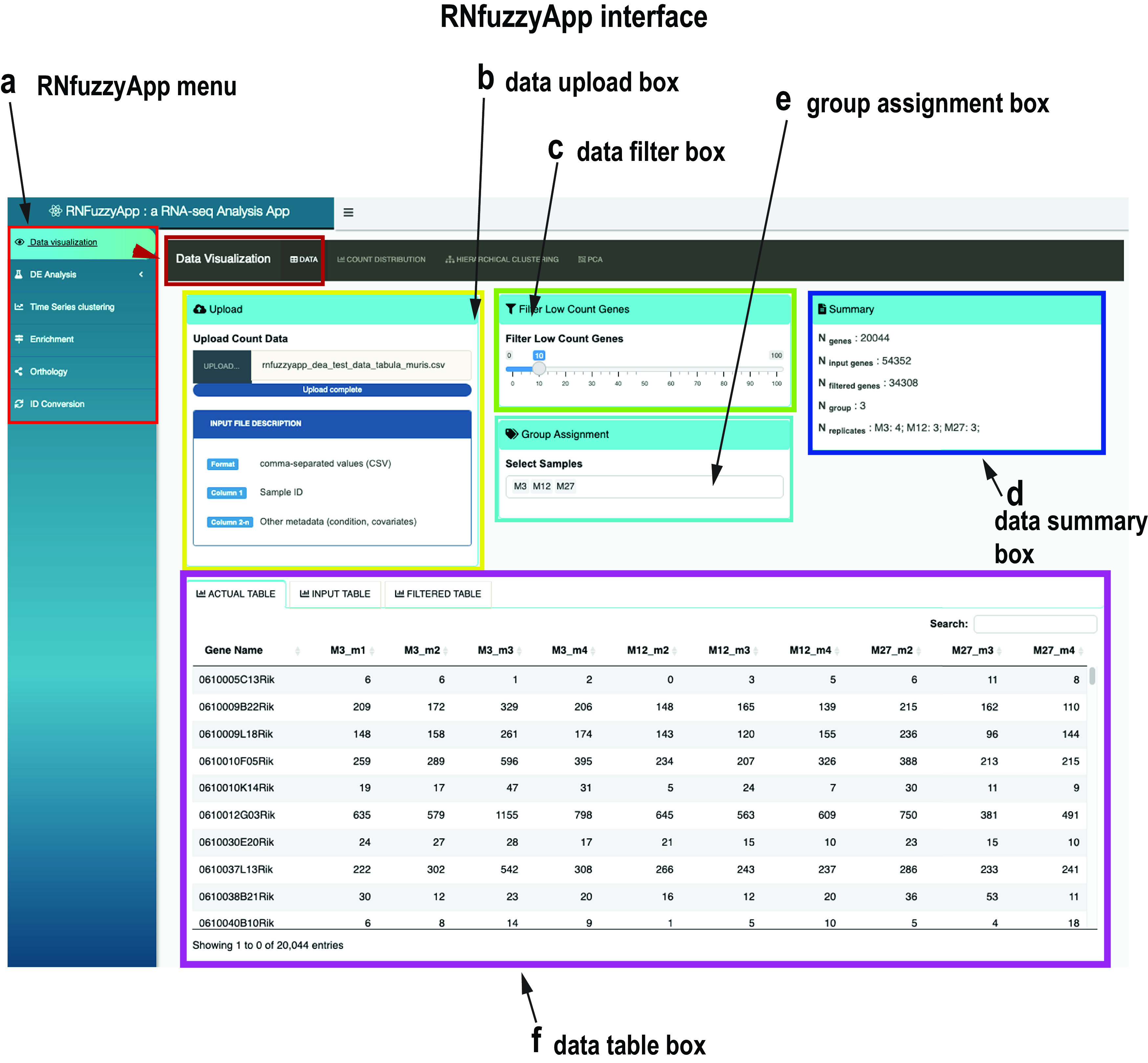
RNfuzzyApp start interface. (a) The RNfuzzyApp menu box is highlighted in red. This box is shown consistently over all interfaces and links to Data visualization, DE analysis, Data normalization and analysis, different visualization possibilities (MA plot, Volcano plot, Heatmap and PCA), Time series clustering (using the pipeline for Mfuzz soft clustering), Enrichment analysis, Orthology assignment, as well as ID-conversion. The main interface shown here belongs to Data visualization (highlighted in dark red). (b) Data upload box, for upload of user-provided data. (c) Data filter box, in which the user can choose to filter out genes with low read counts, (d) in which the identified groups are listed (e.g. wild-type and mutant or different time-points). Group assignment is automatic, so the name format of the samples has to follow a specific pattern. (e) Data summary box. The data shown in this box are updated and in case of filtering and are renewed on the fly. (f) Data table box. Three tables are provided: the actual table, including genes that were not discarded due to filtering; the input table, containing all data uploaded; finally the filtered table, containing genes that were removed due to filtering.

**Figure 3.**
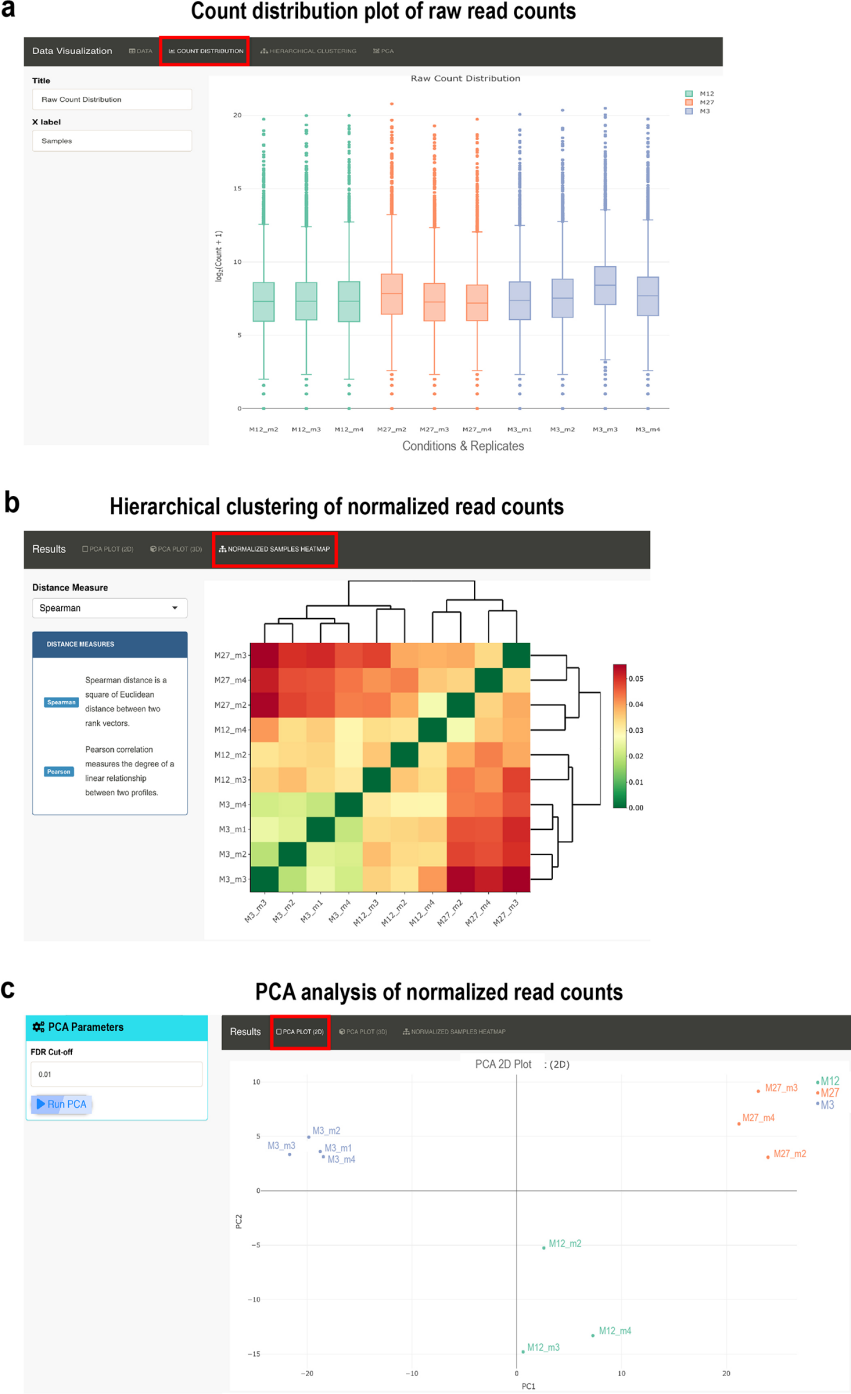
Data visualization plots offered in RNfuzzyApp. We used data from the Tabula muris senis project for demonstration purposes. (a) Count distribution plot of mice from 3 months, 12 months and 27 months. Only replicates from male mice were chosen. Data are grouped by condition. The title of the plot, as well as the X-axis label can be chosen by the user. Raw read counts were chosen for visualization. (b) Hierarchical clustering of normalized read counts. Spearman correlation was used for clustering. (c) PCA plot of normalized read counts. The PCA plot can be visualized in 2D or 3D. DESeq2 was used for normalization of data.

Figure 2 shows the RNfuzzyApp start interface, featuring data upload, filtering, as well as raw data visualization possibilities. As a first step, raw read counts need to be uploaded to the app, in the form of a csv count matrix. Data can be filtered for raw read counts ( Figure 2c), the resulting summary of the data are interactively updated in the Summary box of the interface ( Figure 2d). Groups can be assigned directly in the interface ( Figure 2e). Three tables are available for download: the actual table, containing only the genes that pass the filtering threshold; the original input table; as well as a table containing all genes filtered out due to low read counts ( Figure 2f). Raw data can also be visualized (see top of the menu, Figure 2): the count distribution of raw read counts can be visualized ( Figure 3a). Moreover, raw read counts can be used for hierarchical clustering, as well as a PCA analysis.

*Data normalization and differential expression analysis*

RNfuzzyApp offers several packages for data normalization, as well as for differential expression analysis. Normalization can be done using DESeq2; TMM (trimmed mean of M values), RLE (relative log expression) or upperquantile offered by edgeR; finally the TCC package providing TMM or DESeq2 normalization. As for raw read counts, the count distribution, a heatmap for clustering samples ( Figure 3b), as well as PCA analysis with a 2D as well as 3D PCA plot ( Figure 3c) is available for visualising normalized data. Differential expression analysis can be done using DESeq2, edgeR and bayseq. If more than two conditions are uploaded, normalization and initial differential expression analysis will be done over the entire data set. However, it is often useful to perform pairwise comparison of two conditions or time-points. For pairwise comparisons of two conditions or time-points of larger datasets, a Filter menu is provided. Data resulting from pairwise comparison can be visualized with MA and Volcano plots. All plots are interactive and the user can obtain detailed information about a gene hovering over the dots of the plots. All details on normalization and differential expression analysis can be found in the user manual of RNfuzzyApp.

*Clustering of expression data using heatmaply*

**Figure 4.**
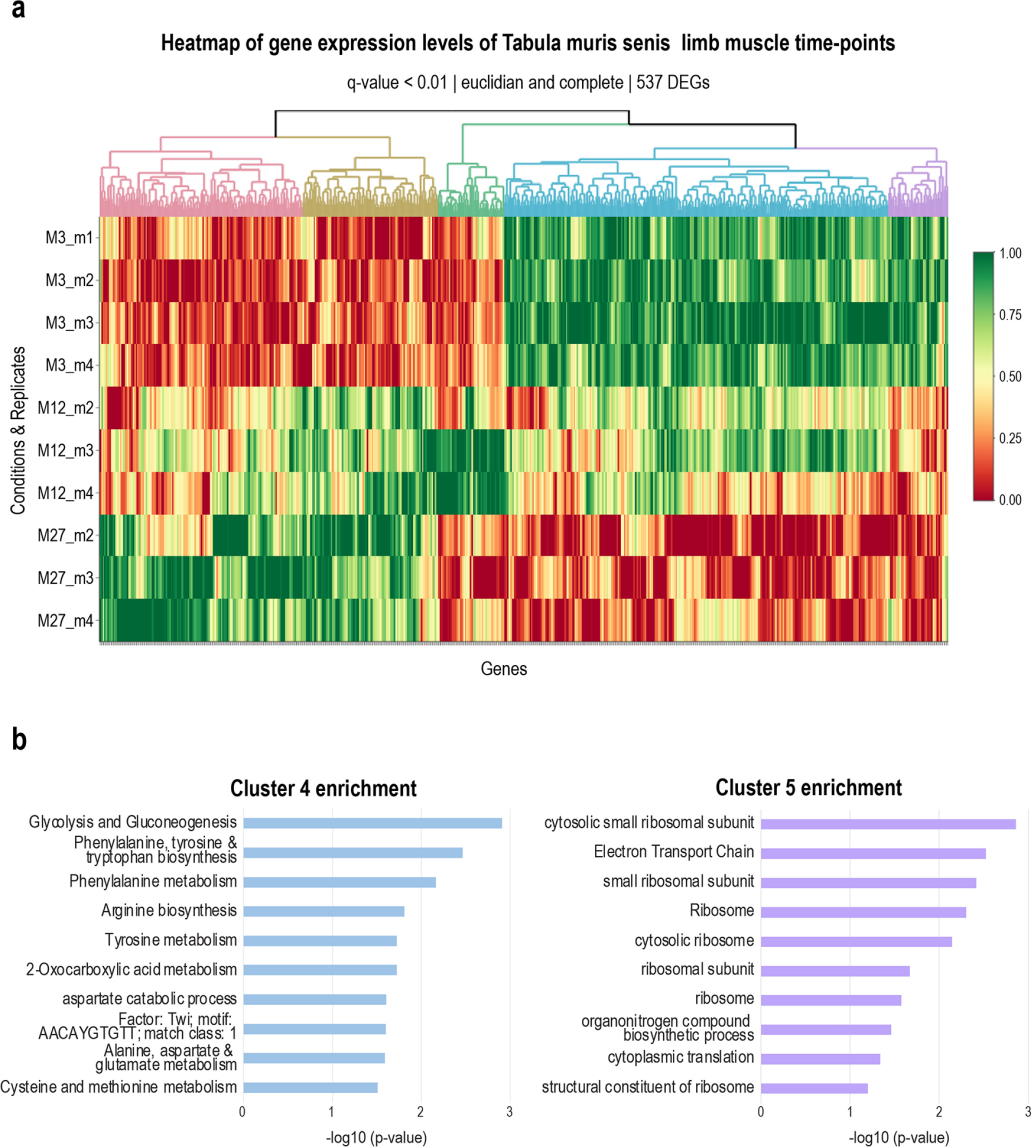
Heatmaply clustering of Tabula muris senis limb muscle data. (a) Heatmap of gene expression levels of the Tabula muris senis limb muscle data. Only significant genes were selected for plotting, with an FDR cutoff of 0.01, resulting in 624 DEGs. These DEGs could be clustered in 5 independent clusters, indicated by different colours in the dendrogram. (b) Enrichment analysis results for cluster 4 and cluster 5 of the limb muscle heat map. In cluster 4, processes related to energy and amino acid metabolism are enriched. Genes belonging to this process have a higher expression level in young versus old mice, suggesting more active metabolism in young muscle cells. In cluster 5, processes related to translation are enriched, whereby associated genes have higher expression levels in young muscle versus old muscle. The minus log10 of the p-value of the enriched process is plotted.

We wanted to provide a simple way of clustering gene expression data from a limited number of samples, e.g. from a short time-series. To this end, RNfuzzyApp offers a heatmap, generated by *heatmaply.* Figure 4a shows the heatmap of a time-course of 3 time points from the Tabula muris senis project, ^15^ where the gene expression levels of the replicates per time point are clustered using hierarchical clustering. The user can choose the distance matrix and agglomeration method, as well as the FDR cut-off of genes to include in the clustering. Clustering is done using *hclust* and *cutree* to generate the coloured dendrogram on the heatmap. The genes contained in the different clusters indicated by the colours in the dendrogram are downloadable as a table for further analysis.

*Enrichment analysis and orthology assignment.*

For enrichment analysis of Gene Ontology (GO-) terms, ^16^ pathways (Wikipathways, ^17^ Reactome ^18^ and KEGG ^19^), Human Protein Atlas, ^20^ CORUM data on protein complexes, ^21^ and TRANSFAC, ^22^ the gprofiler2 package is included in RNfuzzyApp. Results are displayed as an image of overall enrichment, as well as a results table. The table together with a bar plot of the enriched term names sorted according to p-value are downloadable by the user ( Figure 4b, bar plot of enrichments generated from downloaded table). Gprofiler2 is also used to find orthologs in another species of a user-provided list of genes. To this end, the user simply needs to upload a list of genes, and select the original and the target species.

### Complete workflow for fuzzy clustering of time-series data

**Figure 5.**
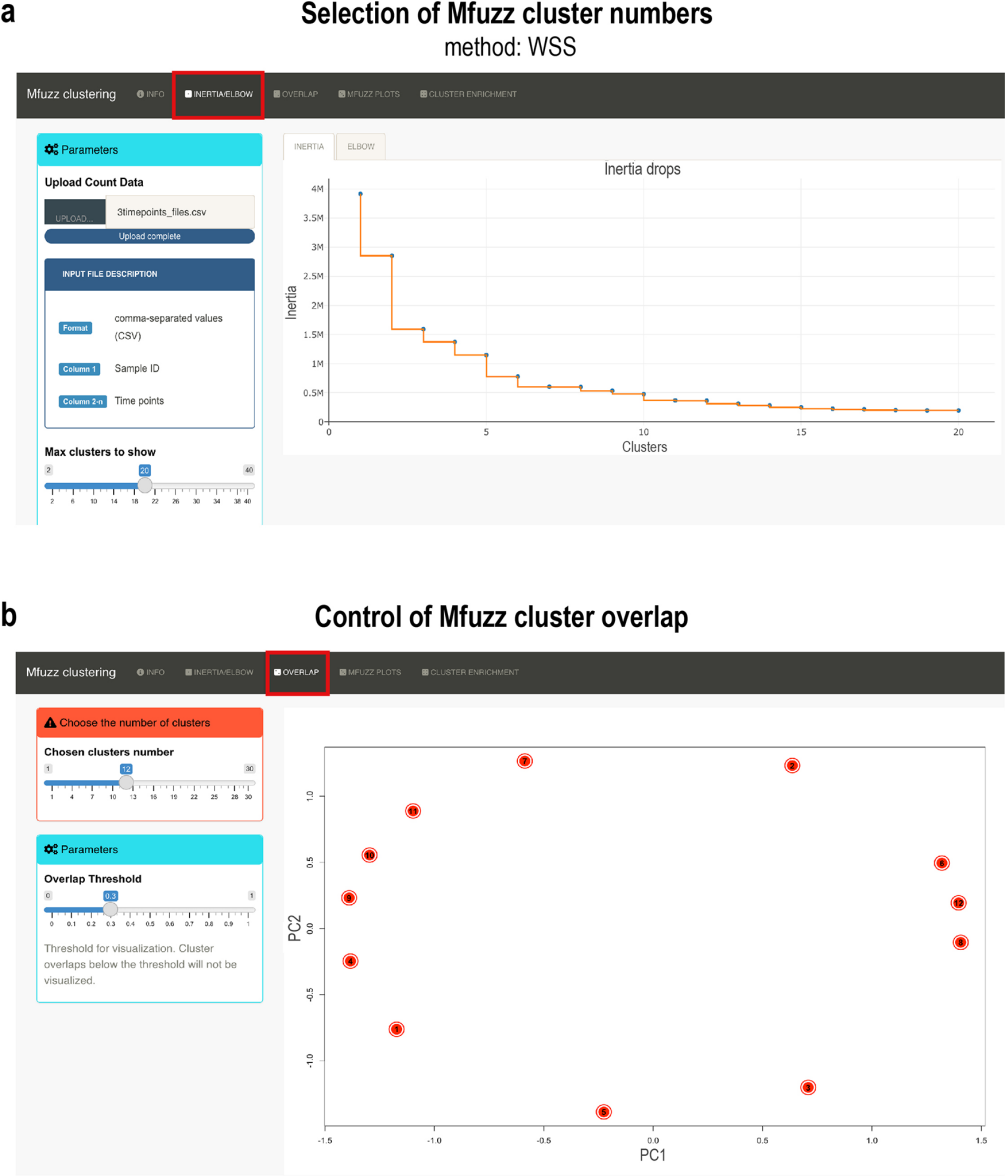
Pre-processing steps required for Mfuzz cluster analysis and cluster number selection. (a) Pre-clustering of data to select the cluster number for Mfuzz time-series clustering. The plot shows the *intertia* drops of the dendrogram. At 12 clusters, the inertia drop was minimal, suggesting that additional clusters would not provide better modelling of the data. (b) Control plot of Mfuzz cluster overlap. A PCA plot is performed with the selected 12 clusters, showing here that no overlap between clusters exists. Data from the developing *Drosophila* leg were chosen for demonstration purposes.

**Figure 6.**
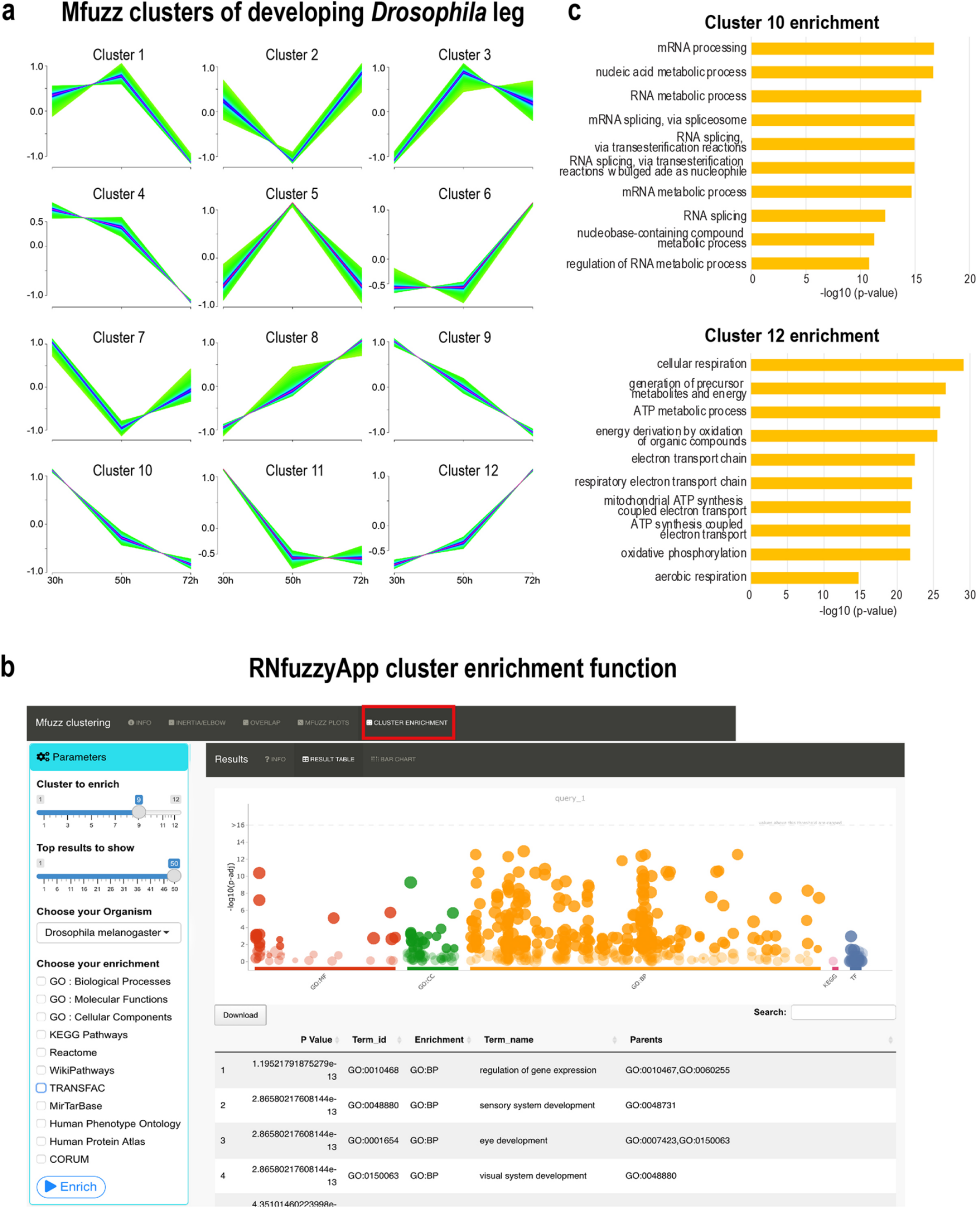
Mfuzz soft clustering analysis. (a) Mfuzz clusters of *Drosophila* leg developmental RNA-seq data. Some similar patterns emerged, with expression profiles peaks early (30 h), mid-phase (50 h) or late (72 h APF (after puparium formation)). (b) Mfuzz clusters have been enriched using gprofiler2. In the parameters box, settings have to be chosen, such as the cluster number submitted for enrichment, the number of results to show, the organism, as well as the databases used for enrichment analysis. The plot on the right-hand side shows the enrichment of cluster 9. (c) Enrichment of the top 10 processes from cluster 10 and cluster 12. In cluster 10, processes related to RNA metabolism, as well as splicing are enriched, associated genes show a decrease in expression over time. In cluster 12, processes related to mitochondrial energy metabolism are enriched, with associated genes showing an increase in expression over time.

Fuzzy clustering of time-series expression data is a highly useful technique for analysing temporal data. The Mfuzz package from R was developed for soft clustering of temporal gene expression data. ^8^ Starting from a count matrix, genes are clustered according to their expression profiles over time. As Mfuzz is a soft clustering algorithm, a gene can in theory be part of more than one cluster. Mfuzz, however, is not straightforward to use for non-experts. First, a number of clusters must be chosen prior to clustering. Second, repeated Mfuzz runs will result in slightly different cluster memberships of genes. A user is therefore well advised to repeat Mfuzz clustering several times to test the robustness of the clustering. The decision, which cluster number is suited for the data then often includes analysis of cluster overlaps, as well as enrichment analyses of clusters and comparative analysis between several chosen numbers of clusters. Several packages exist to help decide on cluster numbers and the entire workflow for a successful Mfuzz clustering can be programmed in R. However, for untrained bench scientists, this is not easily done. We therefore included the complete workflow of Mfuzz soft clustering of time-series expression data in RNfuzzyApp ( Figure 1b): first, for choosing the right cluster number, we implemented the *inertia* (using the *hclust* and *dist* packages) and *elbow* (using the *e1071* package) methods. *Inertia* performs hierarchical clustering and plots the dendrogram, indicating the distance steps (height) against the number of clusters. Ideally, a cluster number is chosen when the drop in height gets minimal ( Figure 5a). The *elbow* method looks at the total of the within-clusters sums of squares (WCSS) as a function of the number of clusters. The “elbow” shape is formed when WCSS is minimal. These two methods should converge to help choose the right number of clusters. After Mfuzz clustering has been performed, the overlap of clusters can be checked. To do so, the *overlap.plot* function from Mfuzz is used and results can be visualized (see Figure 5b). After choosing a suitable number of clusters, Mfuzz is run ten times in a loop to test the robustness of clustering results ( Figure 1b). Membership lists of the ten Mfuzz clustering runs can be downloaded and checked for robustness. Plots are generated using the *mfuzz.plot* function and are also downloadable ( Figure 6a). Should core clusters be unstable, this entire process can be repeated. Finally, enrichment can be done on Mfuzz cluster gene lists, using gprofiler2 ( Figure 6b).

## Data preparation

Tabula muris senis ^15^ limb muscle raw read data (data accessible at NCBI GEO database, ^23^ accession GSE132040) were taken as is and read into RNfuzzyApp for data processing and differential expression analysis. Raw read data from developing leg muscle ^24^ (data accessible at NCBI GEO database, accession GSE143430) were first averaged over replicates before reading them into RNfuzzyApp, as Mfuzz does not accept replicates (available as Habermann, Bianca; Haering, Margaux (2021), extended Datatables).

## Results

### RNA-seq analysis of Tabula muris senis bulk RNA-seq data on the ageing limb muscle

We used data from the ageing limb muscle from the Tabula muris senis project (GSE GSE132040 ^15^). We selected three time-points: 3 months, 12 months and 27 months. We only used samples from male mice. After data upload, we filtered for lowly expressed genes with less than 50 read counts. We then visualized raw read counts of all samples ( Figure 3a). After normalization using DESeq2, we compared samples using hierarchical clustering ( Figure 3b), which showed that replicates cluster together. We also performed PCA analysis and could confirm that samples from the same time-point cluster together ( Figure 3c). We next subjected samples to differential expression analysis using DESeq2, comparing all time-points against each other (see *Extended data*: Tables 1a-c). We found 177 genes differentially regulated between 12 and 3 months, 873 genes differentially regulated between ages 27 and 3 months and 31 genes differentially expressed between ages 12 and 27 months when using an FDR of 0.01 and a log2FC of |0.5|. Enrichment analysis of the lists of differentially expressed genes revealed terms related to translation in young versus adult mice, metabolic and extracellular organisational processes between young and aged mice, as well as between adult and aged mice ( *Extended data*: Tables 1d-f).

We next used hierarchical clustering of genes to identify gene groups changing over time. After finding appropriate points to cut the dendrogram from hierarchical clustering using *heatmaply*, we found five different clusters with differing expression levels of genes ( Figure 4a): two clusters with high expression levels in aged mice and low in young mice, one with high expression levels in adult mice, and two with high expression levels in young mice and low expression levels in aged mice. We subjected all clusters to enrichment analysis ( *Extended data:* Tables 2a-e). For example, cluster 4 and 5, which both show high expression levels in young and low ones in aged mice, had terms related to translation as well as metabolism (energy, amino acids) highly enriched, suggesting more active translation, as well as metabolism in young muscle cells ( Figure 4b).

### Mfuzz soft clustering of a time-series of RNA-seq data from the developing *Drosophila* leg

We used RNA-seq data from a developmental time course of *Drosophila* leg for soft clustering using the Mfuzz pipeline included in RNfuzzyApp. We used normalized read counts from GEO ^25^ gene expression dataset GSE143430 ^24^ and uploaded it to RNfuzzyApp. In brief, leg samples had been collected at three stages during pupal development (30, 50 and 72 h APF) and had been subjected to RNA-sequencing. We wanted to analyse the wild-type expression profiles of genes during these three developmental stages and to identify potentially enriched terms and pathways.

We first checked with the *inertia* method the ideal number of clusters ( Figure 5a). We chose 12 clusters, as we found no significant change with cluster numbers higher than that. We next tested the overlap of clusters using the *overlap.plot* function of Mfuzz and found good separation of the 12 clusters ( Figure 5b). We ran Mfuzz for clustering gene expression profiles and repeated this step 10 times. One of the resulting Mfuzz plots is shown in Figure 6a. We found expression profiles with high expression at 30 h gradually decreasing at 50 h and 70 h (clusters 9 and 10), high expression at 30 h and 50 h, which dereased at 70 h (clusters 1 and 4), expression peaks at 50 h (clusters 3 and 5), low expression levels at 50 h (clusters 2 and 7), low expression levels at 30 h, gradually increasing at 50 h and 70 h (clusters 8 and 12), as well as expression peaks at 70 h (cluster 6). We used all genes of each cluster for enrichment analysis using gprofiler2 within RNfuzzyApp ( Figure 6b, *Extended data:* Table 3a-l). We found terms relevant for muscle development enriched in cluster 10 (high expression at 30 h, which gradually decreased at 50 h and 70 h), relating to mRNA metabolic processes and specifically, RNA splicing. RNA splicing has been shown to be essential for muscle cell type specification ^26, 25^ ( Figure 6c). Cluster 12, which contained genes with increasing expression levels from 30 h to 70 h was enriched for terms related to mitochondrial function and energy production ( Figure 6c). These results are in accordance with earlier observations of increasing electron transport chain components in flight muscle development. ^27, 26^

## Conclusions

We introduced RNfuzzyApp, an intuitive R shiny app for the complete and interactive workflows of RNA-seq, as well as time-course RNA-seq data analysis. RNfuzzyApp includes several algorithms for data normalization and differential expression analysis and offers the possibility for intuitive and interactive data visualization. All data tables and plots are downloadable by the user. While several R shiny apps exist for differential expression analysis, to the best of our knowledge, this is the first web-based, user-friendly R shiny interface for the complete workflow of time-series analysis using the soft clustering app Mfuzz, making RNfuzzyApp the first accessible tool for time-series analysis for wet-lab biologists. We demonstrated the usability of RNfuzzyApp with two examples of RNA-seq data, one from a mouse ageing study of the Tabula muris senis project, and one from the developing leg in *Drosophila melanogaster.*

We chose to offer several packages for normalization, as well as differential expression analysis. This allows the user to exploit several possible combinations of tools for differential expression analysis. Our choice for enrichment analysis in this version of RNfuzzyApp fell on gprofiler2. There are many software tools available for enrichment analysis. Gprofiler2, however, is available also for non-standard model organisms. Therefore, our app can be used for organisms other than human, mouse, *Drosophila*, *C. elegans* or yeast. Moreover, gprofiler2 allows in addition ID conversion, as well as ortholog assignment and both these functions were made available in RNfuzzyApp. In future releases of RNfuzzyApp, we consider including more enrichment tools, providing a broader spectrum of data to include, such as EnrichR. ^28^

To conclude, RNFuzzyApp is an intuitive and easy to use R shiny app that was designed for experimental biologists to enable them to perform RNA-seq and time-series RNA-seq analysis without the need of coding to get a fast overview of their data, results and figures.

## Data availability

### Underlying data

Gene Expression Omnibus (GEO): Tabula Muris Senis: Bulk sequencing. Accession number GSE132040; https://www.ncbi.nlm.nih.gov/geo/query/acc.cgi?acc=GSE132040. ^15^

Gene Expression Omnibus (GEO): Muscle-type specific transcriptomic expression patterns in Drosophila. Accession number GSE143430; https://www.ncbi.nlm.nih.gov/geo/query/acc.cgi?acc=GSE143430. ^24^

### Extended data

Dryad: Extended data tables to Haering and Habermann, F1000Res, RNfuzzyApp: an R shiny RNA-seq data analysis app for visualisation, differential expression analysis, time-series clustering and enrichment analysis. https://doi.org/10.5061/dryad.8pk0p2nnd.

This project contains the following extended data:

• Table 1a: Haering_etal_extendedDatatable_1a_Tabulamurissenis_3vs12m_DEA.txt: results of differential expression analysis (DEA) of Tabula muris senis project (GSE132040), limb muscle, 3 vs 12 months.
• Table 1b: Haering_etal_extendedDatatable_1b_Tabulamurissenis_3vs27m_DEA.txt: results of DEA of Tabula muris senis project (GSE132040), limb muscle, 3 vs 27 months.
• Table 1c: Haering_etal_extendedDatatable_1c_Tabulamurissenis_12vs27m_DEA.txt: results of DEA of Tabula muris senis project (GSE132040), limb muscle, 12 vs 27 months.
• Table 1d: Haering_etal_extendedDatatable_1d_Tabulamurissenis_3vs12m_gpofiler.txt: gprofiler results of Tabula muris senis project (GSE132040), DEA, limb muscle, 3 vs 12 months.
• Table 1e: Haering_etal_extendedDatatable_1e_Tabulamurissenis_3vs27m_gpofiler.txt: gprofiler results of Tabula muris senis project (GSE132040), DEA, limb muscle, 3 vs 12 months.
• Table 1f: Haering_etal_extendedDatatable_1f_Tabulamurissenis_12vs27m_gpofiler.txt: gprofiler results of Tabula muris senis project (GSE132040), DEA, limb muscle, 3 vs 12 months.
• Table 2a: Haering_etal_extendedDatatable_2a_Tabulamurissenis_cluster1_gpofiler.txt: gprofiler results of hierachical clustering of Tabula muris senis project (GSE132040), limb muscle, cluster 1.
• Table 2b: Haering_etal_extendedDatatable_2b_Tabulamurissenis_cluster2_gpofiler.txt: gprofiler results of hierachical clustering of Tabula muris senis project (GSE132040), limb muscle, cluster 2.
• Table 2c: Haering_etal_extendedDatatable_2c_Tabulamurissenis_cluster3_gpofiler.txt: gprofiler results of hierachical clustering of Tabula muris senis project (GSE132040), limb muscle, cluster 3.
• Table 2d: Haering_etal_extendedDatatable_2d_Tabulamurissenis_cluster4_gpofiler.txt: gprofiler results of hierachical clustering of Tabula muris senis project (GSE132040), limb muscle, cluster 4.
• Table 2e: Haering_etal_extendedDatatable_2e_Tabulamurissenis_cluster5_gpofiler.txt: gprofiler results of hierachical clustering of Tabula muris senis project (GSE132040), limb muscle, cluster 5.
• Table 3a: Haering_etal_extendedDatatable_3a_DmLeg_cluster1_gpofiler.txt: gprofiler resuts of mfuzz clustering of Drosophila leg dataset (GSE143430), cluster 1.
• Table 3b: Haering_etal_extendedDatatable_3b_DmLeg_cluster2_gpofiler.txt: gprofiler resuts of mfuzz clustering of Drosophila leg dataset (GSE143430), cluster 2.
• Table 3c: Haering_etal_extendedDatatable_3c_DmLeg_cluster3_gpofiler.txt: gprofiler resuts of mfuzz clustering of Drosophila leg dataset (GSE143430), cluster 3.
• Table 3d: Haering_etal_extendedDatatable_3d_DmLeg_cluster4_gpofiler.txt: gprofiler resuts of mfuzz clustering of Drosophila leg dataset (GSE143430), cluster 4.
• Table 3e: Haering_etal_extendedDatatable_3e_DmLeg_cluster5_gpofiler.txt: gprofiler resuts of mfuzz clustering of Drosophila leg dataset (GSE143430), cluster 5.
• Table 3f: Haering_etal_extendedDatatable_3f_DmLeg_cluster6_gpofiler.txt: gprofiler resuts of mfuzz clustering of Drosophila leg dataset (GSE143430), cluster 6.
• Table 3g: Haering_etal_extendedDatatable_3g_DmLeg_cluster7_gpofiler.txt: gprofiler resuts of mfuzz clustering of Drosophila leg dataset (GSE143430), cluster 7.
• Table 3h: Haering_etal_extendedDatatable_3h_DmLeg_cluster8_gpofiler.txt: gprofiler resuts of mfuzz clustering of Drosophila leg dataset (GSE143430), cluster 8.
• Table 3i: Haering_etal_extendedDatatable_3i_DmLeg_cluster9_gpofiler.txt: gprofiler resuts of mfuzz clustering of Drosophila leg dataset (GSE143430), cluster 9.
• Table 3j: Haering_etal_extendedDatatable_3j_DmLeg_cluster10_gpofiler.txt: gprofiler resuts of mfuzz clustering of Drosophila leg dataset (GSE143430), cluster 10.
• Table 3k: Haering_etal_extendedDatatable_3k_DmLeg_cluster11_gpofiler.txt: gprofiler resuts of mfuzz clustering of Drosophila leg dataset (GSE143430), cluster 11.
• Table 3l: Haering_etal_extendedDatatable_3l_DmLeg_cluster12_gpofiler.txt: gprofiler resuts of mfuzz clustering of Drosophila leg dataset (GSE143430), cluster 12.
• Table 4: Haering_etal_extendedData_DmdevLeg_GSE143430_mean.txt: mean normalized read counts from GSE143430 to be uplaoded for Mfuzz clustering.

Data are available under the terms of the Creative Commons Zero “No rights reserved” data waiver (CC0 1.0 Public domain dedication).

## Software availability

Software available from: https://gitlab.com/habermann_lab/rna-seq-analysis-app.

Zenodo: 10.5281/zenodo.5084275 ( https://zenodo.org/record/5084275#.YO_e_y0iuik).

Source code available from: https://gitlab.com/habermann_lab/rna-seq-analysis-app.

Archived source code as at time of publication: Zenodo: RNfuzzyApp: an R shiny RNA-seq data analysis app for visualisation, differential expression analysis, time-series clustering and enrichment analysis. https://doi.org/10.5281/zenodo.5084275. ^29^

License: GNU public license 3.

## Author contributions

MA and BHH conceived this project. MA was solely responsible for code implementation, software development and testing. MA and BHH performed data analyses. MA and BHH wrote this manuscript.
