# Supplementary material for: RNfuzzyApp: an R shiny RNA-seq data analysis app for visualisation, differential expression analysis, time-series clustering and enrichment analysis
Source: F1000Res. 2021 Nov 12;10:654. Originally published 2021 Jul 26. [Version 2] doi: 10.12688/f1000research.54533.2 (PMC8825645; doi:10.12688/f1000research.54533.2)
Supplement: Peer review history

# Peer review: Reviewer response for version 2

**DOI:** 10.5256/f1000research.79151.r100046

**Published:** 2021-12-07

**Authors:** Rhonda Bacher

**Affiliations:** 1 Department of Biostatistics, University of Florida, Gainesville, FL, USA

All concerns have been addressed.

Are the conclusions about the tool and its performance adequately supported by the findings presented in the article?

Yes

Is the rationale for developing the new software tool clearly explained?

Yes

Is the description of the software tool technically sound?

Yes

Are sufficient details of the code, methods and analysis (if applicable) provided to allow replication of the software development and its use by others?

Yes

Is sufficient information provided to allow interpretation of the expected output datasets and any results generated using the tool?

Partly

Reviewer Expertise:

genomics, RNA-seq, single-cell RNA-seq, time-series RNA-seq, package development, R shiny

I confirm that I have read this submission and believe that I have an appropriate level of expertise to confirm that it is of an acceptable scientific standard.

# Peer review: Reviewer response for version 2

**DOI:** 10.5256/f1000research.79151.r100047

**Published:** 2021-11-15

**Authors:** Oliver Hahn

**Affiliations:** 1 Department of Neurology and Neurological Sciences, Stanford University, Stanford, CA, USA

The authors are commended for addressing all my concerns. I support the indexing of the manuscript!

Are the conclusions about the tool and its performance adequately supported by the findings presented in the article?

Partly

Is the rationale for developing the new software tool clearly explained?

Yes

Is the description of the software tool technically sound?

Yes

Are sufficient details of the code, methods and analysis (if applicable) provided to allow replication of the software development and its use by others?

No

Is sufficient information provided to allow interpretation of the expected output datasets and any results generated using the tool?

Partly

Reviewer Expertise:

Bioinformatics, Systems Biology, Epigenetics

I confirm that I have read this submission and believe that I have an appropriate level of expertise to confirm that it is of an acceptable scientific standard.

# Peer review: Reviewer response for version 1

**DOI:** 10.5256/f1000research.58026.r90441

**Published:** 2021-08-25

**Authors:** Anita Grigoriadis

**Affiliations:** 1 Cancer Bioinformatics, Cancer Centre at Guy's Hospital, King's College London, London, UK

This is an excellent paper presenting an R-shiny to analyze RNA-seq data for non-computational scientists. It provides clear instruction on how to proceed, gives several options to analyze the data, and even includes a mfuzz clustering method, to work with time-series data. I can foresee that it will be used frequently.

 Haering and Habermann have clearly stated why the software is developed. RNAseq analyses has become a standard analytical approach. This software will give non-computational scientists to explore RNAseq data on several levels.

 The description of the software is sound and can be reproduced by others.

 The graphical display and the results provide detailed information, so that the analysis and the data can be interpreted accurately.

Are the conclusions about the tool and its performance adequately supported by the findings presented in the article?

Yes

Is the rationale for developing the new software tool clearly explained?

Yes

Is the description of the software tool technically sound?

Yes

Are sufficient details of the code, methods and analysis (if applicable) provided to allow replication of the software development and its use by others?

Yes

Is sufficient information provided to allow interpretation of the expected output datasets and any results generated using the tool?

Yes

Reviewer Expertise:

cancer bioinformatics

I confirm that I have read this submission and believe that I have an appropriate level of expertise to confirm that it is of an acceptable scientific standard.

# Peer review: Reviewer response for version 1

**DOI:** 10.5256/f1000research.58026.r90440

**Published:** 2021-08-16

**Authors:** Rhonda Bacher

**Affiliations:** 1 Department of Biostatistics, University of Florida, Gainesville, FL, USA

This manuscript presents an R shiny application called RNfuzzyApp. The app is designed for ease-of-use for visualizing and analyzing RNA-seq datasets. The app is very nicely designed and has the potential to be very useful to scientists. However, a number of clarifications are needed.

1. What is orthology assignment mean? This should be defined more clearly in the manuscript
2. It is not clear how to install or run the app. This took me a few tries to figure out. I finally just downloaded the folder from gitlab and then did: > library(shiny)                  > runApp("~/Downloads/rna-seq-analysis-app-master-App/App/"). Instructions should be put on the main page of the Gitlab landing page if that is where users will be initially directed.
3. A simple example csv the users/reviewers can download to test the app would be useful. This would also help users understand the format that the app is expecting. I tried to upload the csv from the GEO and this message appeared: “Maximum upload size exceeded”.
4. Improve error handling, I tried uploading data with genes on the rows and the app crashed saying it had duplicate row names. The app also crashed or would freeze when trying to upload a dataset that was a 5.5MB csv (8 samples). Are there limitations to the upload size?

I highly suggest finding someone to try the app who was not involved in the development to ‘try to break it’ to identify issues like these.

Are the conclusions about the tool and its performance adequately supported by the findings presented in the article?

Yes

Is the rationale for developing the new software tool clearly explained?

Yes

Is the description of the software tool technically sound?

Yes

Are sufficient details of the code, methods and analysis (if applicable) provided to allow replication of the software development and its use by others?

Yes

Is sufficient information provided to allow interpretation of the expected output datasets and any results generated using the tool?

Partly

Reviewer Expertise:

genomics, RNA-seq, single-cell RNA-seq, time-series RNA-seq, package development, R shiny

I confirm that I have read this submission and believe that I have an appropriate level of expertise to confirm that it is of an acceptable scientific standard, however I have significant reservations, as outlined above.

# Response

**Published:** 2021-11-04

**Authors:** Bianca H Habermann

**Affiliations:** Aix-Marseille Univ, CNRS, IBDM, France

We would like to thank Dr. Bacher for reviewing our paper and their very useful comments, which helped us to improve our App, as well as the paper. We have tried to address all points. Indeed, they were greatly overlapping with the comments from Dr. Hahn and show that our user instructions were really done very poorly. We have tried to improve them greatly by creating a very detailed user menu that is available from our gitlab account.

 Concerns raised:

*1. What is orthology assignment mean? This should be defined more clearly in the manuscript*

**Our response:**

 Orthology assignment means that we can find the orthologs of another species using the gprofiler package. We have now tried to clarify in the text what we mean and provide a detailed user manual on how to use this function. 

 2. *It is not clear how to install or run the app. This took me a few tries to figure out. I finally just downloaded the folder from gitlab and then did: > library(shiny)                  > runApp("~/Downloads/rna-seq-analysis-app-master-App/App/"). Instructions should be put on the main page of the Gitlab landing page if that is where users will be initially directed.*

**Our response:**

 We now provide on the gitlab readme page the instructions on how to install the app. We also tried to remove any installation error that we encountered on machines with a very ‘basic’ R environment. We also provide a detailed user manual, which again includes installation instructions and detailed instructions on how to use the app. The user manual is available from the RNfuzzyApp gitlab page.

*3. A simple example csv the users/reviewers can download to test the app would be useful. This would also help users understand the format that the app is expecting. I tried to upload the csv from the GEO and this message appeared: “Maximum upload size exceeded”.*

**Our response:**

 We now provide test files for both functions of RNfuzzyApp , differential expression analysis, as well as Mfuzz clustering, on our gitlab account (in App/test_files).

 The “maximum upload size exceeded error” could be due to size limits within R shiny, which we have tried to circumvent. The size limit of R shiny can be changed with the following command: options(shiny.maxRequestSize = n*1024^2). We have changed this to 100MB, which should be sufficient for most datasets. In case of larger datasets, this would have to be set individually by the user.

*4. Improve error handling, I tried uploading data with genes on the rows and the app crashed saying it had duplicate row names. The app also crashed or would freeze when trying to upload a dataset that was a 5.5MB csv (8 samples). Are there limitations to the upload size?*

**Our response:**

 See our response above: it seems that R shiny has a rather low size limit for data upload, which can be changed with the command: options(shiny.maxRequestSize = n*1024^2). We have set it to 100MB, which should suffice for most datasets.

*5. I highly suggest finding someone to try the app who was not involved in the development to ‘try to break it’ to identify issues like these.*

**Our response:**

 We have tried this now and hopefully have found all obvious bugs of RNfuzzyApp.

# Peer review: Reviewer response for version 1

**DOI:** 10.5256/f1000research.58026.r90442

**Published:** 2021-08-11

**Authors:** Oliver Hahn

**Affiliations:** 1 Department of Neurology and Neurological Sciences, Stanford University, Stanford, CA, USA

RNA-sequencing (RNA-seq) is a widely used technology across a range of biomedical fields. While the method itself - that is, the data generation step - is relatively easy to execute by wet lab researchers or can be outsourced into genomic core facilities, the analysis of the resulting data remains a challenge without substantial coding experience. This is particularly the case for experimental designs involving more than two conditions (i.e. treatment vs. control) as it is the case for time course data or dose-response paradigms. In this study, the authors present a new, graphical user interface (GUI) software package, RNAfuzzy App, with the aim to provide a single environment for standard quality control, normalization, differential expression, clustering and functional enrichment operations.

 The authors re-analyzed two time course datasets from mouse and fruitfly to demonstrate the basic steps and workflows that can be executed with RNAfuzzy App. The package is distributed as a single R shiny app, that the authors design with the intent for easy installation and setup. The demonstrated workflows encompass known steps in RNA-seq analysis including filtering, quality control (via PCA/hierarchical clustering), normalization with several state-of-the-art methods and differential expression involving the accepted software packages Deseq2 and edgeR. The authors highlight the implementation of a soft-clustering workflow involving iterative runs of the Mfuzz algorithm (for analysis of time course trajectories), as well as diagnostic plots that are aiding the user towards correct selection of adjustable workflow parameters. Finally, the authors use the clustering results as input for a functional enrichment workflow, that includes reference data from a range of pathway and transcription factor databases.

 The authors have pursued the development of a highly relevant software package that provides a very comprehensive set of functions and tools. Current RNA-seq data is generated at impressive scale yet remains frequently under-analyzed due to limited coding experience of the experimenters that generate the data. The implementation of elaborative clustering methods and diagnostic plots to aide the user in deciding for the right settings is particularly commendable. Similarly relevant is the implemented functional enrichment workflow, that is well-suited within this package, instead of just finishing the analysis with a set of gene lists that needs to be interpreted elsewhere. The workflows are based on accepted practices and are in agreement with the field’s ‘standard’ analyses. This is important and commendable as several other tools provide misleading workflows that experimental biologists are unaware of.

 However, the software package is currently challenging to install, several functions throw error messages or cause a crash of the app altogether. There is - at least as far as I can tell - very limited documentation (none regarding installation) on how to prepare input data or operate individual workflows. I provide specific details below. I consider myself proficient in bioinformatics in general and RNA-seq data analysis in R in particular, and I struggled and ultimately failed to operate this app.

 While I do acknowledge that the authors are not primarily responsible for the individual challenges the user may face - there are always unforeseeable issues - it needs to be noted that a significant emphasis of the manuscript is on the ‘user-friendly’ (mentioned at least six times in the manuscript) features of RNfuzzy. It also makes the software, which is the heart of this manuscript, difficult to review. There is no link to an already-running ‘reviewer web version’ or a built-in function that loads a tutorial dataset (like the ones used in the manuscript). I have tried to load both the published datasets and a bulk RNA-seq dataset of my own but could not progress beyond loading and quality control due to frequent errors or crashes. That was probably due to some mis-formatting of the input data but without clear guidance on how to prepare the count matrix, or at least an already-formatted dataset, I do not see how untrained experimental biologists will be able to efficiently interact with this app.

 If the authors can improve on these aspects and after I have been able to verify the functionality of the software (at least with data provided by the authors or within a ‘reviewer version’), I would support the indexing of the manuscript.

 In addition, there are multiple points regarding the analysis and statistical methods that would be required to be addressed as well.

 Please find my detailed comments below:

                                                                                                       

 Major concerns:

1. Currently there is no guidance on how to install and launch the app (at least to my knowledge). The authors state on page 3 that ‘Installation instructions are also available.' This is not correct - the adjacent github provides no instructions on how to install the app or how to launch it. Most users do not know how to start a shiny app in their own desktop environment. In addition, when launched, the app throws an error message as the devtools package is not properly loaded and so the ‘install_github’ function is missing. Only when opening R, loading devtools and then launching the app, the setup (including auto-install) is working.
2. There is very limited information on how to prepare the input count matrix. The authors state that a csv matrix is required but there is no information on how genes or sample names should be provided. I assume as row- and column-names, respectively, but that is not clear. Also, should the genes receive an extra header (i.e. a column name that says ‘genes’ or something like that)? Should genes be provided as symbols, ensmebl ids…?
  Neither the github or the app provide much information herein. E.g. the ‘input file description’ within the app states:

  How are all sample IDs provided in just one column? How should other covariates be supplied here? I could not find information on how to use covariates in the provided analysis workflows, neither in the manuscript nor within the app.
  If the input data is not provided correctly, I assume that many analyses will not work or just crash. So this input step is critical. To prevent failures here, the authors could provide a) a detailed description on the input format and/or b) the option to load a template/tutorial dataset.
3. While I was able to load a count matrix into app and run e.g. the hierarchical clustering, all other functions caused errors (e.g. Count distribution, PCA) or a complete crash (normalization, differential expression using DESeq2). I assume this to be due to some problems with the input data, but I cannot verify that as no example/tutorial dataset was provided.

Minor concerns:

1. The authors allow the filtering of low count genes. Could the authors provide references on the legitimacy of this? Packages like Deseq2 require an unaltered count matrix to properly build up e.g. independent filtering, dispersion estimation, etc. and are therefore warranting against any prior filtering or data manipulations.
2. Is the PCA plot calculated on the whole count matrix, or only the filtered one? Is the data somewhat transformed (rlog, varianceStabilizing transformation?) before running the PCA? The PCA function is currently running very slowly and may benefit from some sort of feature selection (e.g. top 10,000 expressed genes).
3. Why do the authors limit their time course analysis to just three timepoints of the published tabula muris datasets? The dataset contains up to 10 timepoints and it may be more convincing to demonstrate the usefulness of RNfuzzy on as many datapoints as possible.
4. The enrichment analysis is currently only allowing ‘Only annotated genes’ or ‘All known genes’ as statistical domain scope (i.e. the ‘background set’). It is common within the RNA-seq field to rather use a set of ‘expressed genes’ to compensate for biases in e.g. tissue-specific expression. DEGs from neuronal tissue will inevitably be enriched for neuronal functions when the whole genome is used as background. To prevent wet lab researches to be deceived by such results, a user-provided expressed set (e.g. normalized counts of 1+ ) or one that is directly loaded from the input matrix, would be beneficial.

Are the conclusions about the tool and its performance adequately supported by the findings presented in the article?

Partly

Is the rationale for developing the new software tool clearly explained?

Yes

Is the description of the software tool technically sound?

Yes

Are sufficient details of the code, methods and analysis (if applicable) provided to allow replication of the software development and its use by others?

No

Is sufficient information provided to allow interpretation of the expected output datasets and any results generated using the tool?

Partly

Reviewer Expertise:

Bioinformatics, Systems Biology, Epigenetics

I confirm that I have read this submission and believe that I have an appropriate level of expertise to confirm that it is of an acceptable scientific standard, however I have significant reservations, as outlined above.

# Response

**Published:** 2021-11-04

**Authors:** Bianca H Habermann

**Affiliations:** Aix-Marseille Univ, CNRS, IBDM, France

We thank Dr. Hahn for their very critical and helpful comments. Indeed, we failed to provide a thorough user manual to help inexperienced users with the installation procedure, as well as the workflow itself. As Dr. Hahn correctly points out - this software should be user-friendly and not challenge the user with problems, already at the installation step. We hope that with the help of Dr. Hahn’s comments, we could significantly improve the user-friendliness of RNfuzzyApp and would like to thank him here for his valuable input.

 In general, we do not provide a running web-server. Our idea was to provide an app that can be locally installed, also because of limitations in server availability on our side. However, we have now tried to significantly improve the installation instructions, as well as corrected several issues of RNfuzzyApp during the installation procedure, which arose due to the fact that many of us make daily use of some of the tools required during automatic installation of RNfuzzyApp. We also realized, when installing it on a ‘brand-new’ machine that has not seen many of the required packages before that quite a few of them need to be installed in addition. We have now added all required packages to the install procedure and thoroughly tested it. We therefore hope that the software installs smoothly. However, it should be noted that depending on the users’ environment, still some errors could occur due to missing pre-installed packages that should normally be present within the R environment. The best advice is to read the error messages during installation within R, which should list the packages that still need installing. 

 Our responses to the **major** and **minor** concerns of Dr. Hahn:

**Major concerns:**

*1. Currently there is no guidance on how to install and launch the app (at least to my knowledge). The authors state on page 3 that ‘Installation instructions are also available.' This is not correct - the adjacent github provides no instructions on how to install the app or how to launch it. Most users do not know how to start a shiny app in their own desktop environment. In addition, when launched, the app throws an error message as the devtools package is not properly loaded and so the ‘install_github’ function is missing. Only when opening R, loading devtools and then launching the app, the setup (including auto-install) is working.*

**Our response:**

 We now provide detailed installation instructions in the user manual that can be found in RNfuzzyApp git repository, including how to install shiny itself (should it be missing), how to call the App and what other errors might occur, e.g. when a Mac is used. We have also tried to remove any error coming from missing packages, so installation should now work in most R environments.

*2. There is very limited information on how to prepare the input count matrix. The authors state that a csv matrix is required but there is no information on how genes or sample names should be provided. I assume as row- and column-names, respectively, but that is not clear. Also, should the genes receive an extra header (i.e. a column name that says ‘genes’ or something like that)? Should genes be provided as symbols, ensembl ids…?*

*Neither the github or the app provide much information herein. E.g. the ‘input file description’ within the app states:*

*How are all sample IDs provided in just one column? How should other covariates be supplied here? I could not find information on how to use covariates in the provided analysis workflows, neither in the manuscript nor within the app.*

*If the input data is not provided correctly, I assume that many analyses will not work or just crash. So this input step is critical. To prevent failures here, the authors could provide a) a detailed description on the input format and/or b) the option to load a template/tutorial dataset*

**Our response:**

 a) We now provide all sample data with the App, in a folder called test_files that can be found at the RNfuzzyApp gitlab site and is downloaded when the software is downloaded.

 b) Covariates was a misleading term, in fact, we only allow read counts (from any type of experiment). We have removed it from the Input File Description.

 c) The genes can be provided as any valid identifier, e.g. Symbols, ENSEMBL IDs etc.

*3. While I was able to load a count matrix into app and run e.g. the hierarchical clustering, all other functions caused errors (e.g. Count distribution, PCA) or a complete crash (normalization, differential expression using DESeq2). I assume this to be due to some problems with the input data, but I cannot verify that as no example/tutorial dataset was provided.*

**Our response:**

 While we cannot tell why RNfuzzyApp crashed, we have now tried the app on several of our machines and were not able to get it crashing during normalization or differential expression analysis. We hope that with the provided input sample data, this error will be solved. We also now show in detail in the user manual how to load the data, normalize it, perform differential expression analysis and visualize the results.

 It should be noted at this point that, when uploading samples from three different conditions or time-points, normalization and initial differential expression analysis will be done over all samples. In order to compare only two conditions, and hence profit from the MA and Volcano plot visualizations, we have now introduced the ‘Filter data’ menu, where the user can choose which conditions (or time-points) to compare.

 We have also simplified the sample assignment. The app now automatically recognizes replicates from a given condition, provided that the header of the columns of the respective data are formatted correctly (condition1_rep1, condition1_rep2, …).

**Minor concerns:**

*1. The authors allow the filtering of low count genes. Could the authors provide references on the legitimacy of this? Packages like Deseq2 require an unaltered count matrix to properly build up e.g. independent filtering, dispersion estimation, etc. and are therefore warranting against any prior filtering or data manipulations.*

**Our response:**

 In fact, in the DESeq2 vignette, filtering is suggested (see the DESeq2 vignette, which states ( http://bioconductor.org/packages/release/bioc/vignettes/DESeq2/inst/doc/DESeq2.html#pre-filtering):

 ​​ *’Pre-filtering*

*While it is not necessary to pre-filter low count genes before running the DESeq2 functions, there are two reasons which make pre-filtering useful: by removing rows in which there are very few reads, we reduce the memory size of the dds data object, and we increase the speed of the transformation and testing functions within DESeq2. Here we perform a minimal pre-filtering to keep only rows that have at least 10 reads total. Note that more strict filtering to increase power is automatically applied via independent filtering on the mean of normalized counts within the results function.’*

 However, the user can choose whether they want to do low read count filtering or not.

*2. Is the PCA plot calculated on the whole count matrix, or only the filtered one? Is the data somewhat transformed (rlog, varianceStabilizing transformation?) before running the PCA? The PCA function is currently running very slowly and may benefit from some sort of feature selection (e.g. top 10,000 expressed genes).*

**Our response:**

 a) PCA analysis of raw read counts is done using all the genes.

 b) PCA analysis of normalized data can be done once differential expression analysis has been performed. In this case, the top genes, as well as the FDR-cutoff can be chosen by the user.

 In both cases, the data are log transformed using the log1p() function.

*3. Why do the authors limit their time course analysis to just three timepoints of the published tabula muris datasets? The dataset contains up to 10 timepoints and it may be more convincing to demonstrate the usefulness of RNfuzzy on as many datapoints as possible.*

**Our response:**

 We have limited our analysis here on only very few time-points for easier understanding of the tool and also to provide small test data files. Indeed, Mfuzz clustering will especially be useful to analyse larger time-series. However, this will inevitably lead to large test data files, as well as large figures. As an example, for our dataset on flight muscle development published in (doi: 10.7554/eLife.34058), we have created 40 clusters from 8 time-points using Mfuzz. Using only three time-points makes the demonstration of the software simply easier.

 We would also like to note that many researchers will only have two conditions to compare using the differential expression analysis function, which is equally useful in RNfuzzyApp. 

*4.* *The enrichment analysis is currently only allowing ‘Only annotated genes’ or ‘All known genes’ as statistical domain scope (i.e. the ‘background set’). It is common within the RNA-seq field to rather use a set of ‘expressed genes’ to compensate for biases in e.g. tissue-specific expression. DEGs from neuronal tissue will inevitably be enriched for neuronal functions when the whole genome is used as background. To prevent wet lab researchers from being deceived by such results, a user-provided expressed set (e.g. normalized counts of 1+ ) or one that is directly loaded from the input matrix, would be beneficial.*

**Our response:**

 While we agree in principle with the idea of taking only the expressed genes as a background list, we would prefer to currently not make this possibility available. We would like to note that many enrichment tools do not provide this functionality and either annotated genes or all known genes from the organism’s genome are used as background lists. I do also think that when comparing DEGs from two conditions of a neuronal tissue (e.g. WT and knock-out), or two time-points from the same tissue, not only neuronal-specific terms will appear, so I do not think that the user will be deceived by the results. In our example of the mouse muscle of mice from 3, 12 and 27 months, for instance, muscle terms are not strongly enriched between the different time-points.
